# Editorial: New strategies for treating fusion-driven sarcomas

**DOI:** 10.3389/fcell.2025.1600133

**Published:** 2025-04-03

**Authors:** Madelyn Espinosa-Cotton, Sean B. Lee, Anand G. Patel

**Affiliations:** ^1^ Department of Pediatrics, Memorial Sloan Kettering Cancer Center, New York, NY, United States; ^2^ Department of Pathology and Laboratory Medicine, Tulane University School of Medicine, New Orleans, LA, United States; ^3^ Department of Oncology, St. Jude Children’s Research Hospital, Memphis, TN, United States; ^4^ Department of Developmental Neurobiology, St. Jude Children’s Research Hospital, Memphis, TN, United States

**Keywords:** sarcoma, fusion oncogene, treatment, epigenetics, intratumoral heterogeneity

Sarcomas are rare connective tissue tumors that resemble a wide range of histologies including bone, fat, muscle, nerve and blood vessels. Among sarcomas, a subset of tumors harbors a fusion oncogene that drives transformation and malignant progression. These fusion-driven sarcomas have proven to be challenging to treat, and overall survival for patients with fusion-driven sarcomas remains low especially for patients with relapsed or disseminated disease. These poor patient outcomes underscore the critical need for a better understanding of the unique molecular and cellular biology of fusion-driven sarcomas, and for concerted efforts to develop therapies targeted against these tumors.

In this Research Topic, we have compiled five articles focused on the biology of fusion-driven sarcoma and on emerging targets for the treatment of these diseases. Each article or review addresses important and emerging concepts in the study of these rare cancers. Importantly, they shed light on molecular pathways that may be exploitable for future therapeutic benefit.

In a review, Gustafson et al. discuss the ongoing challenge of rhabdomyosarcoma, a sarcoma that mimics the morphologic and molecular features of developing skeletal muscle (Skapek et al., 2019). Recent single-cell sequencing efforts have elucidated the surprising degree of embryonal patterning from muscle development that is preserved in malignant rhabdomyosarcoma cells, which have recently been consolidated into a standardized nomenclature (Danielli et al., 2024). While rhabdomyosarcoma cells mimic much of early muscle development, they fail to terminally differentiate, and fusion-positive rhabdomyosarcomas exhibit an unexpected plasticity to convert into a neuronal-like identity (Danielli et al., 2024). Despite these advances, much remains to be uncovered–namely, how rhabdomyosarcoma cells undergo transitions between cell states and how those cell states are regulated. Gustafson et al. present the latest understanding of core regulatory circuitry within both fusion-negative and fusion-positive rhabdomyosarcoma and identify emerging tools to target transcriptional circuits.

In a perspective, Stanton and Pomella present a comprehensive overview of the epigenetic drivers within fusion-driven sarcomas. Most of these entities are driven by translocations of two transcription factors; as such, understanding the dysregulation of core regulatory circuits remains a critical goal for advancing our understanding of this disease. The authors present a holistic view for a translational research approach that integrates discovery-based analysis of clinical samples with experimental modeling to uncover how fusion oncogenes drive epigenetic reprogramming in these cancers. Complementary to this review, a perspective by Ponce et al. focuses on a rare but fascinating subtype of sarcoma driven by CIC-rearrangements. These tumors, which were originally categorized as “Ewing-like tumors” have emerged as distinct entities through exhaustive analysis of patient samples (Antonescu et al., 2017; Specht et al., 2014; Kawamura-Saito et al., 2006). The authors summarize current work and emerging models for studying CIC rearranged tumors, and they single out major barriers limiting progress.

Excitingly, this Research Topic includes two original research articles uncovering new therapeutic approaches to treating fusion-driven sarcomas. Lee et al. test novel agents targeting topoisomerase I in Ewing sarcoma models. While camptothecins analogs, such as irinotecan, are often used in many relapsed Ewing sarcoma regimens (Casey et al., 2009; Raciborska et al., 2013), complex pharmacokinetics and toxicity have been barriers to their utility. Using a novel class of agents called indenoisoquinolines, which are long-lived trappers of topoisomerase I, the authors show that they can achieve growth suppression of Ewing sarcoma models *in vitro* and *in vivo*. In the other article, Parker et al. test the strategy of combining FYN inhibitors, which target a downstream target of the driver SS18:SSX fusion, with histone deacetylase inhibitors in synovial sarcoma. The enhanced therapeutic outcome of the dual targeting approach presents a conceptual framework for targeting fusion oncoproteins via their direct downstream targets.

While this Research Topic has collected a diverse set of articles, it also reinforces important questions about fusion-driven sarcomas and outlines key barriers that limit our ability to study these diseases. While it is clear that fusion oncogenes can drive expression of aberrant gene expression programs, it remains unclear why there is such a tissue and histologic specificity for each fusion. For example, translocations between EWSR1 and FLI1 are typical of Ewing sarcoma, while EWSR1::ATF1 fusions drive clear cell sarcoma. To understand lineage specific tolerance and transformation, well-credentialed experimental models are needed. Recent work developing zebrafish models of VGLL2::NCOA1 driven rhabdomyosarcoma may demonstrate a powerful approach for modeling malignant transformation *in vivo* (Watson et al., 2023). Likewise, induced pluripotent stem cells and somatic gene delivery using electroporation have presented new opportunities to control oncogene activation at varying developmental states (Searcy et al., 2023; Imle et al., 2024). Lastly, our field needs a systematic approach to understand the most effective strategy for targeting fusion oncogenes. There are active efforts to target the oncogenes themselves, which has been a major challenge, or as Parker et al. demonstrate, by using inhibitors against druggable targets directly downstream of the fusion oncogene itself. Collectively, the field of fusion-driven sarcomas is poised to make a long-awaited step forward.
